# Ethnomedicine of the Kagera Region, north western Tanzania. Part 3: plants used in traditional medicine in Kikuku village, Muleba District

**DOI:** 10.1186/1746-4269-8-14

**Published:** 2012-04-04

**Authors:** Mainen J Moshi, Donald F Otieno, Anke Weisheit

**Affiliations:** 1 Department of Biological and Preclinical Studies, Institute of Traditional Medicine, Muhimbili University of Health and Allied Sciences, P.O. Box 65001, Dar es Salaam, Tanzania; 2 Department of Biological Sciences, Moi University, Eldoret, Kenya; 3 Faculty of Development Studies, Mbarara University of Science and Technology (MUST), P.O. Box 1410, Mbarara, Uganda

**Keywords:** Ethnomedicine, Kagera Region, Kikuku village, Euphorbiaceae, Asteraceae

## Abstract

**Background:**

The Kagera region of north western Tanzania has a rich culture of traditional medicine use and practice. Traditional medicines are the mainstay of healthcare in this region and are known to support the management of many illnesses such as malaria, bacterial infections, epilepsy, gynecological problems and others. However, most of the plants being used have either not been documented or evaluated for safety and efficacy or both. This study, the sixth of an ongoing series, reports on the medicinal plants that are used at Kikuku village, Muleba District.

**Methodology:**

A semi-structured questionnaire was used to collect information on the common/local names of the plants, parts of the plants used, diseases treated, methods of preparing the herbal remedies, dosage of the remedies administered, frequency and duration of treatment and toxicity of the medicines. A literature review was carried out for information on the ethnomedical uses of the reported plants.

**Results:**

A total of 49 plant species belonging to 47 genera and 24 plant families were documented. The family Euphorbiaceae and Asteraceae had the highest representation. The plants are used for the treatment of skin conditions (10 plants; 20%), bacterial infections and wounds (14 plants; 28.6%), malaria (14 plants; 28.6%), gastrointestinal disorders (11 plants; 22.4%), gynecological problems including infertility (8 plants; 16.3%), hypertension (5 plants; 10.2%), viral infections (7 plants; 14.3%), chest problems (5 plants; 10.2%), diabetes (3 plants; 6.1%), cancer (2 plants; 4.1%), inflammatory conditions (arthritis, rheumatism), HIV and AIDS, and hernia each treated by 1 plant (3 plants in total; 6.1%). Information obtained from the literature indicate that 25 (51.0%) of the therapeutic claims are supported by laboratory results or have similar claims of ethnomedical use from other countries.

**Conclusion:**

Herbal remedies comprise an important and effective component of the healthcare system in Kikuku village with plants in the families Euphorbiaceae and Asteraceae comprising an important part of plants used in the indigenous healthcare management in the village. Malaria and bacterial infections dominate the list of diseases that are managed using traditional medicines.

## Introduction

The Kagera Region in northwestern Tanzania has a rich culture of traditional medicine practice which has the potential to improve the socio-economic development of the region, especially if the plants being used are evaluated for safety and efficacy. The recovery of knowledge and practices associated with medicinal plants is part of an important strategy that is now being linked to the conservation of biodiversity, the discovery of new medicines, and the bettering of the quality of life of poor rural communities [1].

Whereas there exists a big wealth of medicinal plants in the Kagera region very little has been achieved in their documentation and evaluation for biological activity. Among the few documentation studies done is the study by Chhabra and Mahunnah in 1994 [2], which for the first time documented some of the plants used in this region. The current study is the sixth of a recently renewed initiative to document [3,4], evaluate for biological activity [5-7], and assess how plant genetic resources in the Kagera region can be utilized for the development of an herbal medicines industry and therefore contribute to efforts to reduce poverty. Another study done in the region not too long ago was on the documentation of plants used for the management of HIV and AIDS in Bukoba rural district, in which 75 plants belonging to 66 genera and 41 families were documented [8]. The most recent study reported on the plants used for causing abortion in which proof of the concept was established for 11 out of the 21 plants (52.4%) that were documented [9]. This last study provides strong evidence supporting the strength and authenticity of traditional medicine practice in the Kagera region.

The present study is an ethnomedical documentation of medicinal plants used in Kikuku village, Muleba district, north western Tanzania.

## Methodology

### Description of the study site

**Figure 1:**
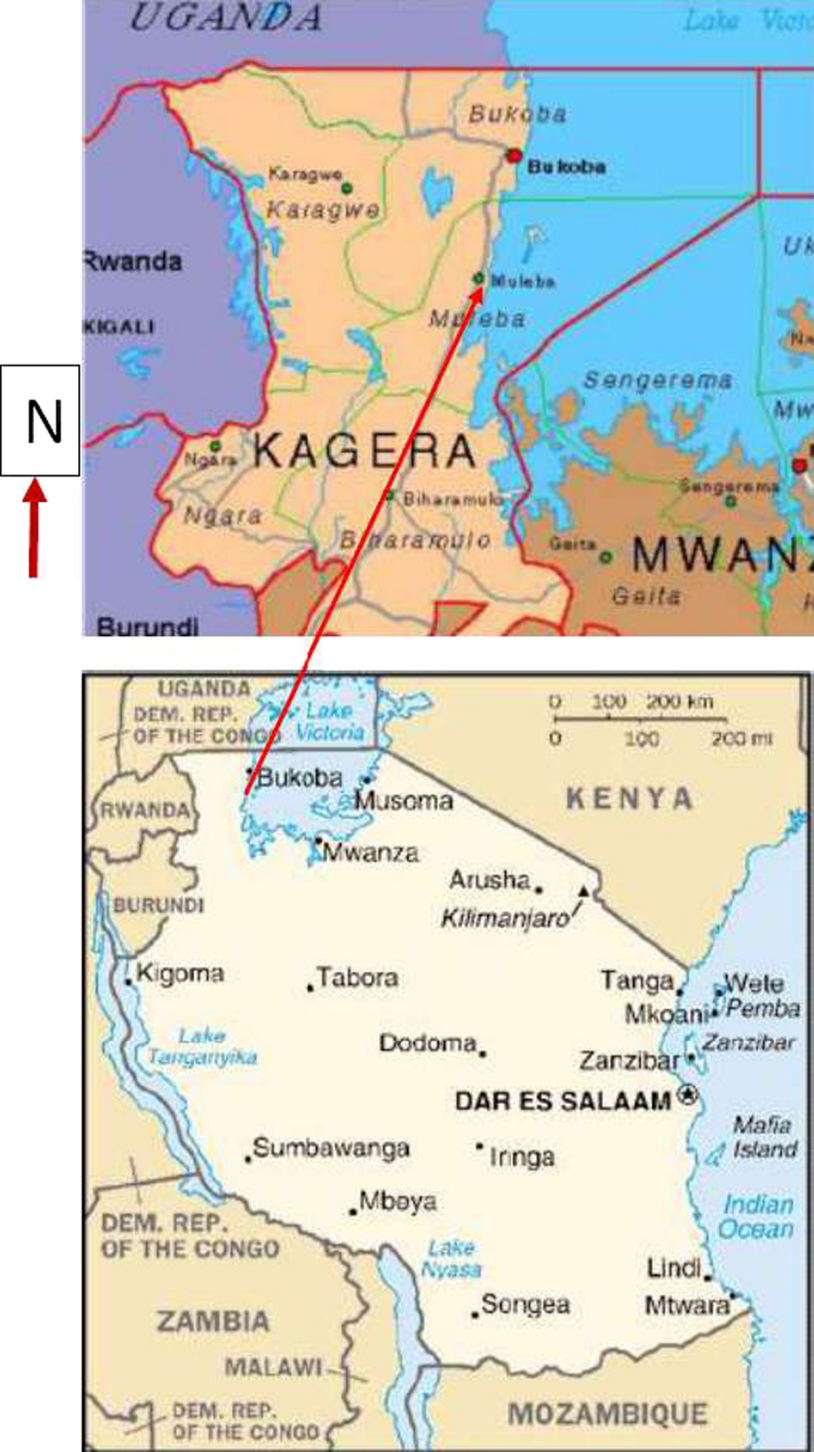
**A map showing the research site at Muleba situated in the northwest part of Tanzania**.

Muleba District lies to the south west of Bukoba town at 1° 50' 23" South, 31° 39' 16" East (Figure 1). It is a second-order administrative division in Tanzania with an average elevation of 1,363 meter above sea level. The area is mildly densely populated with 166 people per sq Km and has a humid ( > 0.65 p/pet) climate. It has a good reserve of medicinal plants as the land area is not cultivated and therefore most of the natural vegetation is still intact. The landscape is mostly covered with closed broad-leaved deciduous forest. It has a tropical savanna climate with a subtropical moist forest biozone. September is on average the month with most sunshine and there is no distinct peak month for rainfall.

### The Ethnobotanical visit and documentation of plant information

Before starting the ethnobotanical visit to Kagera region, one of the research team members travelled to Bukoba to make prior arrangements with the Regional Cultural Office to identify prominent traditional healers in different parts of the region who would be interviewed during our visit. The advance arrangements were done for one week in which period preparations with select traditional healers were made ready for the documentation work. In Muleba District, one informant, a traditional healer practicing at Kikuku village was identified. The Regional Cultural Officers assisted in obtaining prior informed consent from the healer and thus helped to create trust and ease the interview process that subsequently followed. The visit to Kikuku was made on 27th and 28th February and 2nd March, 2008. A semi-structured questionnaire [10] was used to collect information about the plants in an herbal garden within the healer's banana farm, around his house, and in bushes bordering and far away from the farm. Information collected included the common/local names of the plants, parts used, the diseases treated, and methods of preparation, dosage, frequency and duration of treatments. Voucher specimens were made for all plants collected which were subsequently identified by Mr. Selemani Haji of the Department of Botany, University of Dar es Salaam. Duplicate vouchers are kept at the Herbaria of the Botany Department, University of Dar es Salaam and the Institute of Traditional Medicine, Muhimbili University of Health and Allied Sciences.

### Literature survey to establish proof of claims

Literature information was retrieved from the NAPRALERT data base at the School of Pharmacy, University of Illinois at Chicago, and published journal papers were accessed through google, googlescholar and Pubmed. The strength and validity of information obtained from the informant was evaluated based on there being found similar ethnomedical claims in the literature or evidence of laboratory results that support the claims.

## Results

### Medicinal Plant diversity

A total of 49 plant species belonging to 47 genera and 24 plant families were documented (Table 1). The families Euphorbiaceae and Asteraceae with 6 plant species each (24.4%) had the highest representation followed by Fabaceae and Solanaceae (5 plants each, 20.4%). The family Lamiaceae had 3 plant species (6.1%), while Acanthaceae, Amaranthaceae, Apocynaceae, Asparagaceae and Curcubitaceae had two plant species each. The remaining 14 families were each represented by 1 species. The main life forms of the plants used were herbs (38.8%), shrubs (36.7%), trees (16.3%), and climbers (8.2%).

**Table 1:** Medicinal plant families in the study area with the corresponding number of genera and species

Families	Number of genera	Number of species
Acanthaceae	2	2
Amaranthaceae	2	2
Amaryllidaceae	1	1
Apocynaceae	2	2
Asparagaceae	1	2
Asteraceae	6	6
Burseraceae	1	1
Cannaceae	1	1
Cannabaceae	1	1
Chenopodiaceae	1	1
Cleomaceae	1	1
Cucurbitaceae	2	2
Discoraceae	1	1
Euphorbiaceae	6	6
Fabaceae	5	5
Hypericaceae	1	1
Lamiaceae	3	3
Malvaceae	1	1
Phyllanthaceae	1	1
Polygonaceae	1	1
Rubiaceae	1	1
Sapindaceae	1	1
Solanaceae	4	5
Verbenaceae	1	1

The plant parts used for making herbal preparations included roots, leaves, stem barks, root barks, pods, tubers, sap, fruits and other aerial parts. The leaves were the most frequently used part (31 species; 63.3%) followed by the roots and aerial parts (each 7 species; 14.3%), fruits (4 species, 8.1%), stem barks (3 species; 6.1%), tubers (2 species; 4.0%).

### Diseases treated

A wide variety of disease conditions are treated using remedies made from medicinal plants. The diseases for which many different species are used include skin conditions (10 species; 20%), bacterial infections and wounds (14 species; 28.6%), malaria (14 species; 28.6%), and gastrointestinal problems (11 species; 22.4%). Others are gynaecological problems including infertility (8 species; 16.3%), hypertension (5 species; 10.2%), viral infections (7 species; 14.3%), chest problems (5 species; 10.2%), diabetes (3 species; 6.1%), and cancer (2 species; 4.1%). Inflammatory conditions (arthritis, rheumatism), HIV and AIDS, and hernia are each treated using one species (6.1%).

In most cases two or more diseases are treated using one plant. The plant that showed the greatest versatility is *Dracaena steudneri *which is used to treat six different indications. Fourteen species are used to treat one disease condition each (Table 2).

**Table 2:** The medicinal plants of Kikuku village, Muleba District, Kagera Region

Botanical Name [Family] (Voucher Number)	Vernacular name	Life Form	Uses	Part used	Method of preparation and/or administration	Literature reports supporting claims
*Justicia striata *(Ktotzsch) Bullock [ACANTHACEAE] (MJM 3515)	Akalaza	H	Used as an antipyretic	L	The leaves are boiled and the decoction administered to children	

*Whitfieldia elongata *(P.Beauv.) De Wild & T.Durand [ACANTHACEAE] (MJM 3062)	Lugenge/Ekigenge	S	For treating Chicken pox and other skin conditions	AP	Aerial parts squeezed and given to children. The sap also can be applied topically for the treatment of skin conditions or also drank for the treatment of rectal prolapse	Also reported in Bugabo for the same uses [3]

*Amaranthus spinosus *L. [ AMARANTHACE] (MJM 3176]	Olulele	H	For treating peptic ulcers	L	The leaves are boiled with unskimmed cow milk and the preparation drank	Antispasmodic activity [11]

*Dysphanius ambrosioides *(L.) Mosyakin & Clemants [AMARANTHACEAE] (MJM 3178)	Orwita marago/Kaitamarogo	S	For making soap and as a lucky charm	L	The leaves are applied topically.	

*Crinum papillosum *Nodal [AMARYLIDACEAE] (MJM 3236]	Ekiwakye kitunguru	H	For treating swollen breast, legs and abdomen. May also have anti-cancer activity?	TB	The underground tuber is mixed with anthill soil.	Reported to have disinfectant and anti-inflammatory activity [12]

*Catharanthus roseus *(L.) G. Don. [APOCYNACEAE] (MJM3243)	Kiua	S	Treating hypertension	L	The leaves are boiled and the decoction administered	Hypotensive effect of leaves extract confirmed in a rat model of hypertension [13]

*Rauvolfia vomitoria *Afzel. [APOCYNACEAE] (MJM3114)	Omuatabusinde/Kinyabusinde	T	Treating Malaria	L	The leaves are boiled to make a decoction	Reported to have strong antiplasmodial activity [14]

*Dracaena fragrans *(L.) Ker Gawl. [ASPARAGACEAE] (MJM 3128)	Omuramura/Isale	S	To increase CD4 counts in HIV/AIDS patients	R	The roots are boiled with water and a glass taken daily	

*Dracaena steudneri *Engl. [ASPARAGACEAE] (MJM 3169)	Mgologolo	T	For treating hernia, splenomegally, asthma and chest problems in children and fibroids and infertility in women	L	The leaves are burnt and the ash is then combined with soda ash and the powder licked	

*Ageratum conyzoides *(L.) L. [ASTERACEAE] (MJM 3211)	Omwigara	H	Used as a cough remedy, for constipation/peptic ulcers and for fibroids in women or those with difficulties to conceive	R/L	The roots are chewed fresh as an antiacid and antiseptic while the leaves are boiled the decoction taken as tea.	Antiulcer and antispasmodic activity [15-17]

*Dichrocephala integrifolia *(L.f.)Kuntze [ASTERACEAE] (MJM 3188)	Ibuza	H	For treating mouth ulcers and eye infections	L	The leaves are pounded together with those of *Ageratum conyzoides *and the sap squeezed and applied into the eyes as drops.	Antibacterial activity [18]

*Melanthera scandens *(Schumach. & Thonn.) Roberty [ASTERACEAE] MJM 3190]	Byabarwoya	H	For ulcers, wounds and lowering blood glucose levels	L	The leaves are boiled to make a thick decoction which is then drank or the paste applied on a wound	Leaf extracts and fractions inhibited indomethacin, ethanol and histamine induced ulcers in mice [19]

*Microglossa pyrifolia *(Lam.) Kuntze [ ASTERACEAE] (MJM 3175)	Omuhe/Mkuraiju	S	For cleansing airways, treating scalds, cough and flu.	L	The leaves are pounded and the sap is squeezed into the nostrils.	Used for treatment of headaches, and colds [20]

*Vernonia auriculifera *Hiern [ASTERACEAE](MJM 3177)	Kishwiya	S	For treating febrile convulsions	L	The leaves are boiled and decoction administered	

*Senecio stuhlmanii *Klatt [ASTERACEAE] (MJM 3134)	Kikarabo/eirarire	CL H	For treating wounds, swellings, coughs and stiff neck. Also used as an emetic following poisoning	L	The leaves are used for covering containers for making local brews but sometimes are also taken with the local brew. They can also be pounded anded used as an antiseptic dressing. For the treatment of coughs the leaves are baked with s with salt and then chewed	

*Canarium schweinfurtii *Engl. [BURSERACEAE] (MJM 3036)	Muubani	T	For treating malaria and syphilis	SB	The bark is ground into powder and boiled. An aromatic gum is produces which can then be burnt and used as a scent.	Essential oil has antimicrobial activity [21]

*Canna indica *L. [CANNACEAE] (MJM 3172)	Amarango/Embakyo	H	For the treatment of infertility in men and women. It makes a woman conceive easily	L	The leaves are dried, groud and the powder is then used or pounded, soaked in a small amount of water and a patient given a spoonful.	Used in Argentina and China for regulation of fertility [22,23]

*Trema orientalis *(L.) Blume [CANNABACEAE] (MJM 3212)	Muge	T	For treating yellow fever and as a haematinic	AP	The leaves are pounded, then boiled with the leaves of *Combretum collinum *and *Erythrina abbyssinica*. The resulting decoction is used to treat yellow fever. An infusion made from the leaves is also drank as an haematinic	Reported to be used for treatment of chickenpox Nigeria [24]

*Chenopodium opulifolium *Schrad. ex W.D.J. Koch & Ziz [CHENOPODIACE AE] (MJM 3158)	Omwetango	H	For treating venereal diseases and mouth ulcers	L	The leaves are boiled and the decoction drank. The decoction can also be used for bathing	

*Cleome gynandra *(L.)[CLEOMACEAE] (MJM 3239]	Eshobyo/Mgagani	H	For treating arthritis and rheumatism	L	The leaves are eaten as vegetables	Methanol extract of leaves exhibited anti-inflammatory activity in rat adjuvant arthritis [25]

*Cucurbita maxima *Dúchense [CUCURBITACEAE] ( MJM 3168)	Mwongu/boga	H	Epixstasis, excessive menstrual bleeding	FR	The fruit is baked under ashes and squeezed into the nostrils/vagina	

*Zehneria scabra *Sond. [CUCURBITACEAE] (MJM 3150)	Akabindizi	CL.	For treating skin diseases, gonorrhea, syphilis, cleansing uterus before a child is delivered and malaria	L	The leaves are boiled and decoction drank. Often is mixed with several other plants for treatment of malaria	Ethanol extract of tubers exhibited antibacterial activity against gran negative bacteria [26,27]

*Dioscorea praehensilis *Benth. [DIOSCORIACEAE] (MJM 3246)	Amasoma	Cl.	For hypertension and diabetes	TB	The tubers are eaten as food	

*Bridelia micrantha *(Hochst.) Baill.[EUPHORBIACEAE] (MJM 3166)	Mshamako	T	For yellow fever, malaria, amenorrea and dysmenorrhea	R	Roots are boiled with water to make decoction which is drank.	Antimalarial activity [28] and antibacterial activity [29]

*Euphorbia hirta *L.[ EUPHORBIACEAE] (MJM 3184)	Kahyebulimbe empango	H	For non-lactation, hypertension, warts and cataracts (local application)	AP	The aerial parts are mixed with *Solanum nigrum *and boiled and the decoction given to mothers to enhance lactation. Large amounts are sometimes drank to induce diuresis. It is also considered a very safe galactagogue	Reported to have galactogenic activity [30-34]

*Euphorbia mossambicensis *(Klotzsch & Garcke) Boiss. [EUPHORBIACEAE] (MJM 3185)	Kahyebulimbe Enkye	H	As a galactagogue and for treating cataracts	L	The leaves are boiled and the decoction administered orally. The decoction can also be applied to the eye to treat cataracts.	

*Jatropha curcas *L.[ EUPHORBIACEAE] (MJM 3187)	Ekiho	S	For mastitis and koilonichia	L	The leaves are baked under fire, and the sap squeezed into the affected area for 5 days. It should never be taken orally	Antibacterial activity [35-38]

*Ricinus communis *L. [EUPHORBIACEAE] (MJM3142)	Kijuna	H	For skin diseases and cancer	L, R	The leaves are burnt and the ash wetted with water is applied vtopically to the affected area.	

*Tragia furialis *Bojer [EUPHORBIACEAE ] (MJM 3186)	Omugonampili	T	For malaria, cancer and peptic ulcers	L.	In the treatment of cancers, the leaves are burnt in an earthen pot, and the smoke directed to the affected area. A decoction of the leaves in combination with those of other plants is used for treatment of malaria. The leaves are boiled with milk and taken for the treatment of peptic ulcers	

*Abrus precatorius *L. [FABACEAE] (MJM 3013)	Kaligaligo	CL	For malaria	L	The leaves are pounded and boiled with water. The resulting decoction is administered orally.	An isoflavanquinone, abruquinone B, isolated from aerial parts exhibited antiplasmodial activity [39]

*Cajanus cajan *(L.) Millsp. [FABACEAE] (MJM 3226)	Entandaigwa	S	For poisoning, nausea and swelling of legs	AP	The aerial parts are burnt and the ash taken with water for the treatment of poisoning The leaves are boiled with water and one glass of the decoction obtained is taken daily for the treatment of nausea during pregnancy. The leaves are also used for massaging swollen legs.	Ethanol extract exhibited hepatoprotective activity against galactosamine-induced hepatitis in rats [40]

*Dolichos kilimandscharicus *Taub. [FABACEAE] (MJM 3247)	None	H	For malaria, gonorrhea and syphilis	L	The leaves boiled together with other plants	

*Sesbania microphylla *E.Phillips & Hutch.[FABACEAE] (MJM 3171)	Msenga/Mbondo	S	For malaria and febrile convulsions in children	L	The leaves are boiled together with the leaves of *Orthosiphon suffrutescens *and *Solanum aculeastrum *and the decoction administered to children	

*Senna occidentalis *(L.) Link [FABACEAE] (MJM 3179)	Omwetanjoka	S	For spasms, malaria and as an antihelmintic	R	The roots are pounded and then boil with water and natural salt. The resulting decoction is then drank for de-worming. A decoction of the leaves is used to treat malaria.	Antimalarial activity [41,42], and antispasmodic activity [43]

*Harungana madagascariensis *Lam. ex Poir [HYPERICACEAE] (MJM 3119)	Omujumbojumbo	T	For yellow fever and as an anthelminthic	SB, SD	The stem bark is soaked in water or or dried, pounded and the resulting powder is taken with tea The seeds are dried and then powdered. The powder is taken orally for the treatment of all types of worm infestations	Reported to have antihepatotoxic activity [2,44]

*Tetradenia urticifolia *(Baker) Phillipson [LAMIACEAE] (MJM3125)	Omushunshu	H	For ulcers, tonsils, wounds, malaria and insect bite	L	The leaves are boiled to make a decoction which is then drank or pounded and used for dressing. For malaria the leaves are boiled with water and a glass of the decoction taken three times a day.	

*Orthosiphon thymiflorus *(Roth) Sleesen [LAMIACEAE] (MJM 3182)	Lwamo	H	For malaria and febrile convulsions	L	The leaves are boiled and the decoction given to children who are sometimes also bathed in it. The leaves are mixed with those of *Solanum aculeastrum*)	Used for malaria treatment in Rwanda [45]

*Plectranthus barbatus *var. *grandis *(L.H.Cramer) Lukhoba & A.J.Paton [LAMIACEAE] (MJM 3132)	Akajera akake	S	As an antidote for insect bites	AP	Aerial parts are crushed and rubbed on the affected area	

*Hibiscus fuscus *Garcke [MALVACEAE] (MJM 3070)	Omusingasinga	S	For polio	L	The leaves are pounded and mixed with fat (Ghee) and massaged on the affected area.	Extracts of the plant exhibited antiviral activity [46]

*Flueggea virosa *(Roxb. ex Willd) Royle [PHYLLANTHACEAE] (MJM 3183)	Mturuka	S	For gonorrhea and skin conditions	L	The leaves are boiled and the decoction administered orally or applied topically.	Used to treat skin conditions and has proven antifungal activity [47,48]

*Oxygonum cordofanum *(Meisn.) Dammer [POLYGONACEAE] (MJM 3061)	Akanyunyambuzi/Akaikutukura	H	For wounds and warts	L	The leaves are burnt and the ashes licked	

*Tricalysia *coriacea (Benth.) Hiern [RUBIACEAE] (MJM 3173)	Omwani Kibira	S	For skin diseases, epixstasis and malaria/ yellow fever (jaundice)	L, R	The leaves/roots are boiled and the decoction drank	

*Cardiospermum halicacabum *L. [SAPINDACEAE] (MJM 3180)	Oluzibula	S	To treat people with urinary obstruction	L	The leaves are boiled to make a decoction.	

*Capsicum frutescens *L. [SOLANACEAE] (MJM3170)	Obuguluma/pilipili	H	As a cough remedy	L	The leaves are chewed	Decoction is used in Nicaragua as cough remedy [49]

*Cyphomandra crassifolia *Kuntze [SOLANACEAE] (MJM 3117)	Mtomatoma/mtomasi	T	As a cardiotonic, haematinic or for septic rashes in children. Also used to lower blood sugar levels	FR	The fruits are eaten as food to increase blood. The dried seeds are pounded into powder and the powder taken for the treatment of heart condition (cardiotonic)	

*Physalis peruviana *L. [SOLANACEAE] (MJM 3147)	Ntuntunu	H	For typhoid fever	FR	The fruits are squeezed and the juice is used for treatment of typhoid	

*Solanum nigrum *L. [SOLANACEAE] (MJM 3174)	Shwiga	S	For de-worming (hookworms) and treating ring worms and warts. Also used for blood pressure and preventing children from bed wetting	LFR	For the treatment of ringworms the leaves are pounded and applied topically. The leaves can also also be pounded and baked under fire before being used for dressing a wart. The ripe fruit is used to treat bed wetting	Reported to have hypertensive activity and has shown hypotensive effect in the dog [50,51]

*Solanum aculeastrum *Dunal [SOLANACEAE] (MJM 3138)	Omulembezi/Entobatobe	S	For malaria, febrile convulsions and goitre	FR, L	The fruits are placed under ash fire and then applied to the affected area. It is also used to rub the adder of a cow to stimulate milk production. It is mixed with the leaves of *Orthosiphon suffrutescens*) and *Sesbania sesban *for treatment of febrile convulsions	

*Clerodendrum myricoides *R.Br. [VERBENACEAE] (MJM 3198)	Ekisheke	S	For malaria, febrile convulsions, abdominal colics and used for making soap and as a lucky charm	SBRL	The stem bark is boiled with water and half a teaspoonful of the decoction is administerd orally. It can also be applied topically. as a lucky charm or as soap	Root extracts showed invitro activity against Plasmodium falciparum [52,53]

Key: *S *shrub, *T *Tree, *H *herb, *CL *climber, *CL-H *Climber herb: *SB *Stem bark, *R *roots, *L *leaves; *AP *aerial parts, *FR *fruits, *SD *seeds *TB *Tubers

### Methods used in the preparation of herbal medicines

Medicines used were prepared mainly by boiling to make decoctions (40.5%), pounding to paste (14.49%), squeezing (8.69%), chewing (8.69%), soaking in water to make infusions (8.69%), burning to ash (7.24%), grinding to powder (7.24%) and baking under hot ashes (5.79%). Some of the methods were used in combination e.g. grinding bark and then boiling to make a decoction, burning leaves and then mixing with margarine to form paste amongst others (see Table 2 for other examples). The making of decoctions was carried out mostly by boiling the plant parts in water. In three cases they were prepared by making concoctions i.e. boiling more than one plant. In the treatment of yellow fever a concoction is made from the leaves of *Trema orientalis *which are pounded and boiled with the leaves of *Combretum collinum *and *Erythrina abyssinica*. Similarly, a concoction for the treatment of malaria and febrile convulsions is made by boiling the leaves of *Sesbania macrophylla *with the leaves of *Orthosiphon suffrutescens *and *Solanum aculeastrum*. In a third example the leaves of *Orthosiphon thymiflorus *are mixed with those of *Solanum aculeastrum *and boiled; or the leaves of *Solanum aculeastrum *are boiled with *Orthosiphon suffrutescens *and *Sesbania sesban *for the treatment of malaria and febrile convulsions. In the case of *Amaranthus spinosus*, milk instead of water, is used.

### Efficacy of herbal remedies used in traditional medicine

Among the 49 plants used in Kikuku as herbal medicines, 25 (51.0%) were found to have similar ethnomedical claims or have been phytochemically or pharmacologically proven to be active in literature. Those with proven phytochemical or pharmacological activity included plants used for the treatment of malaria e.g. *Bridelia micrantha *[28,29], *Senna occidentalis *[41,42], and *Clerodendrum myricoides *[52,53]. Other reports from the literature are the disinfectant and anti-inflammatory activity of *Crinum papillosum *[12], and hypotensive effect of *Catharanthus roseus *extracts in rats [13]. *Rauvolfia vomitoria *has been confirmed to have a strong antiplasmodial activity [14], while *Melanthera scandens *leaf extract inhibited indomethacin, ethanol and histamine induced ulcers [19] in confirmation of the traditional medicine uses. The use of *Microglossa pyrifolia *for the treatment of headaches and colds has been reported by another source [20], while for *Canarium schweinfurthii *the antimicrobial activity has been confirmed by laboratory results [21]. The use of *Trema orientalis *for treatment of viral infections has been reported elsewhere [24], while the anti-inflammatory activity of *Cleome gynandra *is supported by anti-inflammatory activity of a methanol extract of the leaves against adjuvant-induced arthritis in rats [25]. Other proven claims are the hepatoprotective effect of *Cajanus cajan *ethanol extract in rats [40] and the antimalarial activity of *Abrus precatorius *[39] and *Clerodendrum myricoides *[52,53]. *Orthosiphon suffrutescens *does not yet has proof of the concept laboratory results, but it is used in Rwanda for the treatment of malaria [45]. Evidence from the literature supports the use of *Amaranthus spinosus *[11], *Senna occidentalis *[43], and *Ageratum conyzoides *[15-17] for the treatment of peptic ulcers or as antispasmodics. Other ethnomedical claims supported by the literature include plants used in the treatment of bacterial infections; including *Dichrocephala integrifolia *[18], *Jatropha curcas *[35-38] and *Bridelia micrantha *[29]. The use of *Flueggea virosa *for the treatment of skin conditions is well supported by its confirmed antifungal activity [47,48]. Other plants in this study with ethnomedical claims supported by literature-based evidence are *Canna indica *[22,23], *Capsicum frutescens *[49], *Euphorbia hirta *[30-34], *Harungana madagascariensis *[2,44], *Hibiscus fuscu*s [46], *Solanum nigrum *[50,51], and *Zehneria scabra *[26,27].

## Discussion

Many of the plants used in Kikuku ethnomedicine belong to the families Euphorbiaceae and Asteraceae. The Euphorbiaceae comprises 317 genera and about 8,000 species [54,55], making it one of the largest plant families. It consists of some very useful medicinal plants, such as *Euphorbia Kansu *[55], which probably explains their prominence in the ethnomedicine of the Kikuku people. Since time immemorial many Euphorbiaceae have been popular as medicinal plants [55] and their recorded presence in this study just goes further to buttress this fact. The diverse medicinal properties of the Euphorbiaceae are thought to be associated with its wide range of habitats which predispose plants in this family to high mutation loads (accruing from stressful habitats) and a large range of environmental stimuli making them to develop a wide array of defensive secondary metabolites [55]. Of the approximately 250,000 species of known flowering plants, nearly one in ten are members of the Asteraceae, a diverse family found in almost every habitat on all continents except Antarctica [56]. This fact could probably then explain the prevalence of the Asteraceae, alongside the Euphorbiaceae, in the ethnomedicine of the Kikuku people.

The finding in this study that herbs and shrubs are the predominant life-forms used as medicines is consistent with other reported studies [57,58]. The popularity of herbs as a source of herbal therapies is often attributed to their high pharmacologically active components compared to woody plant forms [59] whilst shrubs seem to be preferred due to the fact that their availability all year round as they are relatively drought resistant and are not affected by seasonal variations [60]. Some of the plants reported in this study (e.g. *Ageratum conyzoides, Amaranthus spinosus, Bridelia micrantha, Capsicum frutescens, Clerodendrum myricoides, Draceana steudneri, Dicrocephalus integrifolia, Euphorbia hirta, Ricinus communis, Senna occidentalis*, and *Zehneria scabra*) have, variously, been found to have similar or different ethno-medicinal uses elsewhere [57,60-65]. This can be considered to be a reliable indication of their pharmacological effectiveness having been tested in different areas by different cultures [60].

This study shows that leaves, followed by roots and other aerial parts are preferred in the preparation of treatment recipes. It is tempting to speculate that practitioners of traditional medicine in Kikuku are conservation conscious and thus mainly use leaves so that they can sustain their supply of this herbal resource. However, on the converse, use of leaves could also be a severe threat to some rare and slowly reproducing medicinal plants [66]. Although the idea that herbalists in Kikuku are aware of the importance of conservation is demonstrated in the widespread establishment of home gardens, it would still be premature to arrive at such a conclusion. More studies have to be done to confirm this assertion. Nevertheless, the use of leaves as a preferred source of traditional medicines has also been reported elsewhere [67]. Their preference to other plant parts is commonly thought to be due to their accumulation of active principles like inulins, tannins and other alkaloids [68].

A total of thirty three different diseases were treated using herbal remedies. This diversity in diseases treated is an indication that medicinal plants have a real potential of meeting the varied healthcare needs of residents of a rural village like Kikuku. Medicinal plants have been shown to be the base of healthcare systems in many societies [1] and therefore their use in the treatment of a wide range of diseases as elucidated in this study goes further to show their centrality in meeting healthcare needs of rural populations. Most of the disease conditions cited by our informant (60.6%) were treated using more than a single species. Malaria, like in previous studies done in Kagera region [3,4], was treated with the highest number of different plants. The use of such a wide variety of plants for the treatment of one disease alone could be an indication that this disease is prevalent in the study area [1]. It could also be due to the availability of a wide variety of herbal medicines indicated for the treatment of the disease [69]. It is not surprising that there were a comparable high number of plants that were used for the treatment of bacterial infections given that these are commonplace indications largely associated with personal hygiene and care. From the results, it seems like malaria and bacterial infections constitute the biggest proportion of health problems common in this part of Tanzania.

Decoctions were cited as the most commonly used dosage form. In the majority of cases it involved directly boiling the part to be used (e.g. leaves) in water or first pounding the part and then boiling as in the case of *Senna occidentalis *where the roots were pounded before boiling. Boiling is considered to be effective in extracting plant materials besides also preserving them longer compared to using cold water [70]. However it also has its drawbacks in that if careful attention is not paid to the time needed for boiling some ingredients may be damaged, especially with prolonged boiling and useful aromatic ingredients present may also be lost through dissipation into the air [71]. Our results show that making of concoctions to treat a given ailment was not a common practice with the healer we interviewed. She only treated three diseases using concoctions. An interesting method of preparation was that of *Amaranthus spinosus *leaves which were boiled with fresh cow milk and drank as a remedy for peptic ulcers.

Close to 43% of the curative claims concerning plants used as traditional medicines in Kikuku were well supported by literature based evidence. This finding is consistent with two previous ethnomedical studies carried out in the Kagera Region [3,4]. The correlation of the traditional uses of some of the plants with their known phytochemical and pharmacological properties lends credence to some of the ethnomedical claims.

## Conclusion

This study shows that the Euphorbiaceae and Asteraceae are important in the indigenous healthcare management in Kikuku village given that they emerge as the families with the highest number of species used as traditional medicine. Some of the correlations observed between traditional plant use in Kikuku village with scientifically proven phytochemical and pharmacological properties of the plants and also with reports of similar use elsewhere, suggests that herbal remedies constitute an important and effective component of the healthcare system in Kikuku village and whose use therefore needs to be encouraged and promoted. However more in-depth phytochemical and pharmacological studies are necessary to support the use of the plants documented in this study.

## Competing interests

The authors have no competing interests in the project, and share the aspirations of the local people of Muleba ward to bring good healthcare services to their community.

## Authors' contributions

MJM, DFO, AW, carried out the design of the study, which is being implemented in Kenya, Tanzania and Uganda. MJM organized the study visit, preparations, interviewed the traditional healers in Kikuku, Muleba District, compiled the information, analyzed and synthesized the manuscript to the final draft. DFO checked and corrected all the Latin binomial names of the plants and contributed to data analysis. All authors read, revised and approved the final manuscript.
